# Beneficial Effects of Interactive Physical-Cognitive Game-Based Training on Fall Risk and Cognitive Performance of Older Adults

**DOI:** 10.3390/ijerph17176079

**Published:** 2020-08-21

**Authors:** Kochaphan Phirom, Teerawat Kamnardsiri, Somporn Sungkarat

**Affiliations:** 1 Department of Physical Therapy, Faculty of Associated Medical Sciences, Chiang Mai University, Chiang Mai 50200, Thailand; kochaphanph@gmail.com; 2 Department of Digital Game, College of Arts, Media, and Technology, Chiang Mai University, Chiang Mai 50200, Thailand; teerawat.k@cmu.ac.th

**Keywords:** fall risk, cognitive function, combined physical and cognitive training, exergaming, health promotion, preventive medicine

## Abstract

Physical and cognitive declines are significant risk factors for falls. Promising evidence suggests that combined physical-cognitive training would be an effective fall risk reduction and cognitive improvement intervention. However, a limited number of studies have been conducted and findings have been inconclusive. This study investigated the effects of interactive physical-cognitive game-based training on the fall risk and cognitive performance of older adults. Forty participants were randomly allocated to the intervention (n = 20) and control (n = 20) groups. Participants in the intervention group performed a 1 h session, 3 times a week for 12 weeks of the interactive physical-cognitive game-based training program. Fall risk (Physiological Profile Assessment, PPA; and Timed Up and Go, TUG) and cognitive outcome (Montreal Cognitive Assessment, MoCA) were assessed at pre- and post-intervention. Thirty-nine participants (mean age = 69.81 ± 3.78 years) completed the study (97.5%). At the end of the trial, participants in the intervention group demonstrated significant improvement in the PPA fall risk score (*p* = 0.015), postural sway (*p* = 0.005), MoCA score (*p* = 0.001), and TUG-dual task (*p* = 0.045) compared to controls. In conclusion, the interactive physical-cognitive, game-based training was effective in reducing physiological fall risk and improving cognitive function in community-dwelling older adults.

## 1. Introduction

Approximately one-third of community-dwelling older adults aged over 65 years suffer a fall each year and the rate of falls increases with age [1]. Physical injuries and the psychological consequences of falls can lead to loss of independence, disability, poor quality of life, and mortality in older people [1,2]. Age-related physical and cognitive declines are common intrinsic causes of falls in older adults [3]. Poor balance, lower limb muscle weakness, decreased reaction time, and visual and proprioception impairment are known factors associated with falls in older adults [4]. Not only physical impairments, declines in cognitive function including executive function, attention, memory, visuospatial ability, and processing speed are also associated with an increase in fall risk and rates of falling [5,6].

Systematic reviews and meta-analyses have consistently reported that physical exercise, especially that which includes a balance component, is an effective fall prevention strategy for older people [7,8]. However, the current body of evidence on the significant role of cognition on balance, gait, and falls suggests that adding a cognitive component to physical exercise may potentially enhance the efficacy of the exercise program for fall prevention [5]. In line with this proposition, previous studies demonstrated that the combined physical-cognitive training leads to significant improvement in physical and/or cognitive function [9,10,11]. Moreover, a few studies found that combined physical-cognitive training results in superior cognitive and physical performance than either type of single training alone, suggesting the synergistic effects of the combined training [11,12,13]. Nevertheless, some studies did not find additional effects of the combined physical-cognitive training on either cognitive performance or fall risk in older adults [14,15]. Such non-positive effects have been attributed to the use of separate training between physical and cognitive components (i.e., sequential training), and the high task demand of the combined training in previous studies. It is suggested that optimal physical and cognitive task demand and the simultaneous training of physical and cognitive components (i.e., dual training) may be required to reveal the positive effects of the combined training [14,15]. Given that there have been a limited number of studies investigating the effects of the combined physical and cognitive training and findings have been inconclusive, further study in this aspect is warranted.

Low compliance and high drop-out rates have often been reported in exercise training programs and partly accounted for non-positive outcomes of the intervention [16]. Currently, game-based exercises (exergames or exergaming) have received increasing attention in supporting healthy aging and fall prevention as a means to enhance exercise adherence. An interactive experience while moving the body in response to the game provides motivation and enjoyment, resulting in a high compliance rate [17,18]. Moreover, exergames allow concurrent physical and cognitive exercise (dual tasking). Recent systematic reviews have demonstrated that exergames improve the physical health (i.e., balance, mobility, physical fitness, and walking performance/gait parameters) and mental health (i.e., executive functions, reaction time, and processing speed) of older adults [19,20,21]. Game-based exercises using the Nintendo, Wii Fit, and Xbox Kinect devices have consistently demonstrated positive effects on balance in healthy older adults. Therefore, exergames have been proposed as a form of fall prevention exercise for the elderly [22,23]. Among various game technologies, the Xbox Kinect sensor has better features than other technologies as it uses 3D depth sensing technology to detect motion without the need of a controller device, thus allowing the free movement of participants in different directions and positions [17]. This study aimed to evaluate the effects of an interactive physical-cognitive game-based training program on fall risk and cognitive function of older adults. To overcome the shortcomings of previous studies, the simultaneous format of combined training and graded task demand was implemented in the form of a game-based training via the Xbox Kinect.

## 2. Materials and Methods

### 2.1. Design and Participants

This study was a pseudo-randomized assessor-blind controlled trial. The sample size in this study was calculated based on the following assumptions: A general linear model, medium effect size (0.24), a power of 0.80, and an alpha level of 0.05. The effect size of 0.24 was obtained from the Physiological Profile Assessment (PPA) fall risk score in the pilot study. With this, a total sample size of 34 participants was required. To accommodate the 15% drop-out, a total sample size of 40 participants (20 participants per group) were recruited in this study.

Eligible participants were community-dwelling older adults aged 65 years or older, able to walk without a walking aid for at least 10 m, and able to step unassisted in different directions safely. Exclusion criteria were major cognitive impairment (Mental State Examination T10 < 23 or depending on the level of education), had depressive symptoms defined as a score more than 6/15 points on the Thai Geriatric Depression Scale (TGDS), history of neurological diseases (e.g., Parkinson Disease, stroke), other health problems affecting stepping ability (e.g., acute painful joint inflammation, mobility impairment), or any unstable health conditions that preclude exercise. All participants gave written informed consent prior to enrollment in the study and were asked the demographic data including age, gender, weight, height, and medication used. The study protocol was approved by the Human Ethical Review Board of the primary investigator’s institution (approval number: AMSEC-61EX-078). Eligible participants were allocated to either the intervention or control groups.

### 2.2. Intervention

**Figure 1:**
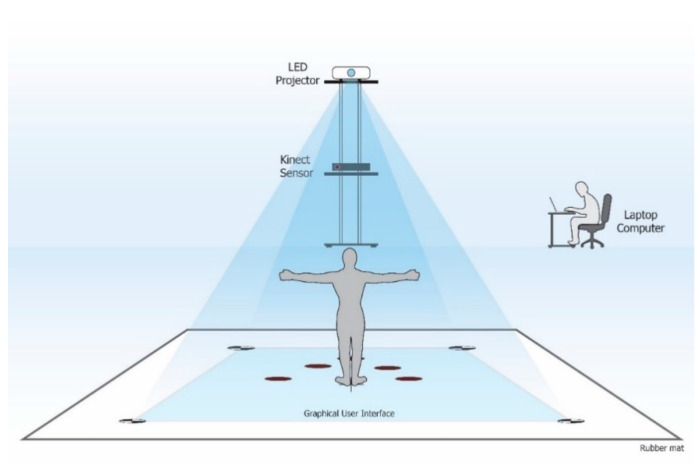
Setting of an interactive game-based training program.

**Figure 2:**
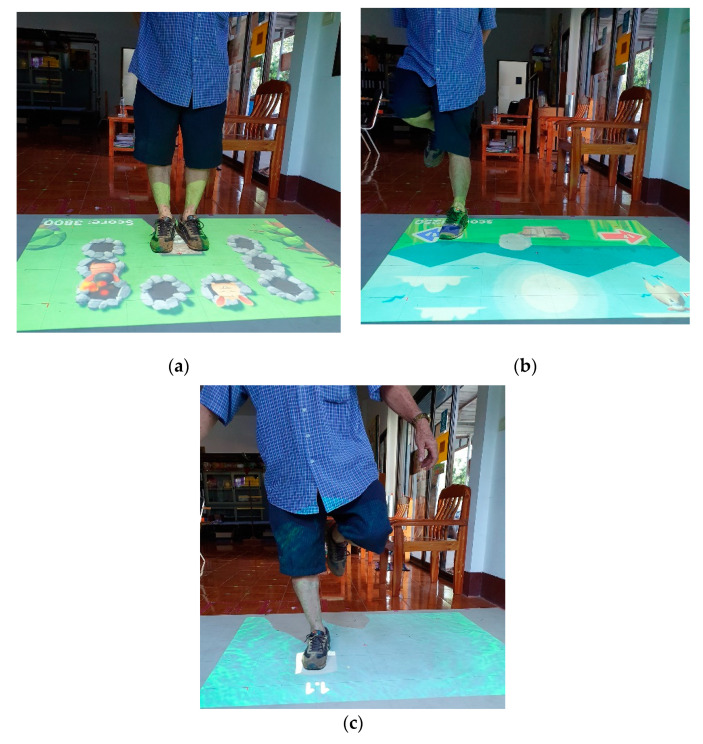
Example of the interactive physical-cognitive game-based training program: (**a**) Whack a mole game; (**b**) Sky fall game; (**c**) Crossing poison river game.

Participants in the intervention group practiced an interactive game-based training program, which simultaneously engaged in the cognitive and physical functioning for 60 min per session, 3 sessions per week for 12 consecutive weeks at their local community centers such as the subdistrict health promotion hospital or village health club. The interactive physical-cognitive game-based settings comprised three main devices including a Microsoft^®^ Xbox 360 Kinect sensor V2, LED projector, and laptop computer (Figure 1). The virtual game was projected to the rubber mat placed on the floor, allowing the participants to interact with the game by stepping on different targets and in different directions in response to the game’s rules. The three-dimensional data of the body collected from the Kinect sensor were used to detect movements and positions of the participants. The interactive physical-cognitive game-based training program was developed by integrating the existing evidence in the literature on the effective physical and cognitive training programs for fall prevention and the exergames used in older adults [24,25,26,27]. There were two main components for the physical parts; stepping on different targets and in different directions, and balance training. As for the cognitive part, there were five games that covered three neurocognitive subdomains (i.e., executive function, attention, and memory) associated with balance and falls in older adults [5,6]. A summary of the cognitive domains included in the combined physical-cognitive training program is described in Table 1. The developed program comprised five games (i.e., Fruits Hunter, Where am I? Whack a mole, Sky fall, and Crossing poison river) challenging in both physical and cognitive ability (Figure 2). The game-based training program had 3 levels of difficulty: Low, moderate, and high, based on both physical and cognitive demands. Movement parameters including speed, distance, duration, or base of support were used to increase the level of physical demand while cognitive complexity including number of stimuli, amount of attention, planning, and memory load was used to increase the level of cognitive demand. Training progression was provided individually based on the participant’s performance. Participants were progressed to the next game level when they scored 80/100 points or more in the current level.

Participants in the control group received educational material covering cognitive enhancement and fall prevention strategies. They received a telephone call from the researcher once a week to monitor their general health and activities. The same information was obtained from the intervention group. Participants in both groups were asked to maintain their routine lifestyle throughout the study period and to inform the research team in case there were any routine changes or unexpected events (e.g., illness, hospital admission, falls).

### 2.3. Outcome Measures

#### 2.3.1. Fall Risk Outcomes

Physiological Profile Assessment (PPA) and Timed Up and Go test (TUG) were used to indicate fall risk. The PPA is a validated tool for quantifying fall risk by assessing five physiological factors associated with falls including postural sway, reaction time, muscle force, proprioception, and vision [4]. The intraclass correlation coefficient (ICC) for five sub-tests of PPA ranged from 0.50 to 0.97, which indicated moderate to excellent test–retest reliability [4]. In the present study, all the equipment used for PPA assessment was standard tools (NeuRA FallScreen^®^, Neuroscience Research Australia, Sydney, Australia) developed by Lord et al. [4]. Postural sway was measured by using a Lord’s sway meter to measure displacements of the center of mass (COM) while standing still on a medium-density foam with eyes open for 30 s. Sway path was recorded in square millimeters (mm^2^). Reaction time was assessed by using a hand-held electronic timer. A light was the stimulus and a finger press on the mouse was the response. Five practice trials were undertaken, followed by 10 testing trials. An average of the 10 testing trials was recorded in milliseconds (ms). Maximal contraction of the knee extensor was used to represent muscle force. Participants were seated with the hip and knee joint at 90° flexion. They were asked to extend their leg against the spring gauge connected to a strap placed 10 cm above the ankle joint for 3 trials. The maximal force of the 3 testing trials was recorded in kilograms (kg). Knee proprioception was measured using a lower limb-matching task. Participants were seated with eyes closed and asked to align their lower limbs simultaneously on each side of an acrylic sheet inscribed with a protractor. The difference in alignment of the lower limbs was measured in degrees. After two practice trials, an average of 5 testing trials was recorded. For vision, the ability to identify the lowest contrast sensitivity was assessed by using the Melbourne Edge Test (MET). Participants were asked to identify the orientation of 20 circular patches with edges of reducing contrast. Correct identification of the orientation of the edge on the patches provided a measure of contrast sensitivity in decibel units (dB). The PPA composite score (standardized score) was calculated from these five weighted subtests through the NeuRA FallScreen^®^ (https://fbirc.neura.edu.au/fallscreen). The lower PPA composite scores indicate lower fall risk. A fall risk is designated mild if the score is between 0 and 1, moderate between 1 and 2, and marked for scores >2 [4].

The Timed Up and Go test (TUG) was used to measure functional mobility fall risk. The TUG is widely used as a screening test for falls in older adults. The TUG test was a validated test to assess the risk of falls and has been reported to have excellent inter- and intra-tester reliability (ICC = 0.97–0.99) [28,29] with a standard error of measurement of 0.7 s in the geriatric population [30]. The time taken to stand up from a standard-height chair, walk at their maximum but safe pace for 3 m, and then turn and walk back to the chair and sit down was measured. Participants received standardized instructions and demonstration. A practice trial was allowed for familiarization prior to data collection. The average time to complete the test in 2 trials was recorded by using a stopwatch. A time greater than 14 s indicates risk of falls [28]. In addition, the TUG with a verbal fluency task was also assessed to indicate dual-task ability. The TUG with the dual-task test consisted of the TUG combined with the verbal attention-demanding task of the naming task (animal and fruit naming task in the first and second trial, respectively). The naming task was based on a well-established verbal fluency test [31], which has been used as a component in various dual-task tests [32,33]. The participants were asked to perform the TUG test in concurrence with the naming test. The average time to complete the test in 2 trials was recorded for the TUG single and dual task.

#### 2.3.2. Cognitive Outcome

The Montreal Cognitive Assessment (MoCA) was used for the global cognitive evaluation. The MoCA is a valid and reliable test (ICC = 0.81) to assess cognitive performance in a healthy population aged 55 years and older [34]. The Thai translated version of the MoCA has been shown to be valid and reliable (Cronbach’s alpha = 0.74) [35]. The MoCA is a simple cognitive screening tool that assesses 8 cognitive domains including (1) attention and concentration, (2) executive functions, (3) memory, (4) language, (5) visuo-constructional skills, (6) conceptual thinking, (7) calculations, and (8) orientation [36]. Attention and concentration were evaluated using a sustained attention task and digits forward and backward. Executive function was assessed using an alternation task adapted from the trail-making B task. Memory was assessed using a recall task involving two learning trials of five nouns and delayed recall. Language was assessed using a naming task with low-familiarity animals, repetition of two complex sentences, and the aforementioned fluency task. Visuo-constructional skills were assessed using a clock-drawing task and a three-dimensional cube copy. Conceptual thinking was assessed using a two-item verbal abstraction task. Calculation was assessed using a serial subtraction task (calculation task). Finally, orientation was evaluated by asking the participants for the date and the city in which the test is occurring. The time needed to administer the MoCA is approximately ten minutes. The total possible score is 30 points. One point was added to the score for an educational level equal to or less than 12 years of formal education. The higher score reflects a better global cognitive function [36].

All outcomes were assessed at baseline and the end of the 12 weeks at the Department of Physical Therapy, Faculty of Associated Medical Sciences, Chiang Mai University by trained assessors, blinded to the participant’s group allocation. Structured instructions and demonstration were provided to the participants before testing. A five-min rest period was given between each test to minimize the influence of fatigue.

### 2.4. Data Analysis

The Shapiro–Wilk test was used to determine data normality and the analysis demonstrated that all variables were normally distributed. Descriptive statistics were used to describe the demographic characteristics of the participants. The independent samples t-test (for continuous data) and chi-square test (for categorical data) were used to determine differences between the intervention and control groups at baseline. A two-way mixed-model ANOVA was used to compare the outcome measures across the two different assessment intervals (at baseline and the end of the 12 week period) and between the two groups (intervention and control groups). Bonferroni was used for the post-hoc analysis in this study. Partial Eta-squared values were reported as measures of effect size. SPSS software (version 21.0, IBM Corporation, Chicago, IL, USA) was used for all statistical analyses. The alpha level was set at 5%.

## 3. Results

### 3.1. Baseline Characteristics

Seventy-four participants were screened. Twenty-three participants did not meet the inclusion criteria (i.e., cognitive impairment (n = 3), health conditions that affect exercise ability (n = 6), education less than 4 years (n = 3), age less than 65 years old (n = 9), use assistive device (n = 2)) and eleven participants were unwilling to participate in the study. Forty eligible participants were enrolled and randomized to either the intervention group (n = 20) or control group (n = 20). Thirty-nine participants completed the study, which corresponded to an overall drop-out rate of 2.5%. One participant in the training group could not attend the re-assessment, due to health reasons unrelated to the study. Participants in the intervention group attended on average 35.6 ± 0.8 sessions (98.8%) and no adverse events were reported. The demographic characteristics of all participants are illustrated in Table 2. There were no significant differences between groups in any demographic characteristics at baseline.

### 3.2. Effects of Combined Physical-Cognitive Training on Fall Risk

Fall risk as measured by the PPA composite score, and TUG-single and TUG-dual task is shown in Table 3. Mixed-model repeated-measures ANOVA revealed significant interaction effects of group × time for the PPA composite score (*p* = 0.002) and its subcomponents including postural sway (*p* = 0.001) and hand reaction time (*p* = 0.010). Post-hoc analysis identified that at the end of the training period, the PPA composite score (*p* = 0.015) and postural sway (*p* = 0.005) were significantly lower for the intervention than that of the control group (Table 3). Group analyses revealed that at the end of the training period, participants in the intervention group had significant performance improvement as revealed by a decrease in PPA composite score (*p* = 0.001), reaction time (*p* = 0.024) and postural sway (*p* = 0.003), while control participants demonstrated a significant performance decline as revealed by an increase in postural sway (*p* = 0.025) from baseline. In addition, there were significant group × time interactions for the TUG tests in both the single (*p* = 0.015) and dual task (*p* = 0.025). After the 12 week period, participants in the intervention group took a significantly shorter time to perform the TUG-dual task than controls (*p* = 0.045). Their TUG performance in both the single and dual task after training was also significantly better than baseline (*p* = 0.001), while such improvement was not observed for the control group.

### 3.3. Effects of Combined Physical-Cognitive Training on Cognitive Function

Cognitive performance of the combined physical-cognitive training and control groups is illustrated in Table 4. Mixed-model repeated-measures ANOVA revealed significant interaction effects of group × time for the MoCA score (*p* = 0.001) along with its subtests including executive function, attention, language, and abstraction (all *p* < 0.05). At the end of the 12 week trial, participants in the intervention group demonstrated significantly better performance on the MoCA. Furthermore, subtest analyses revealed that they had significantly higher scores on executive function (*p* = 0.003), attention (*p* = 0.001), and abstraction subtests of MoCA (*p* = 0.009) when compared to controls at the 12 week period. Group analyses showed that participants in the intervention group demonstrated significant improvements in attention (*p* = 0.005) and language subtests (*p* = 0.040) at the end of the training period compared to baseline. By contrast, participants in the control group demonstrated significant performance declines from baseline for the attention (*p* = 0.020) and abstraction subtests of MoCA (*p* = 0.008).

## 4. Discussion

Findings from this study demonstrated that the 12 week-combined physical-cognitive training program decreased fall risk and improved cognitive performance. The significant decrease in PPA composites score after training indicated the beneficial effect of the program on fall risk. Further analyses of the PPA sub-components revealed that the reduction in PPA composites score was primarily due to the improvement in postural sway and reaction time performance. The present findings are in agreement with a previous study that demonstrated improvement in reaction time, postural sway, and PPA composite scores in older adults after participation in the home-based step pad training using videogame technology [10]. The faster reaction time after training, which suggested a faster central processing speed, was likely due to the game characteristics that emphasized the choice stepping response. During the interactive physical-cognitive game-based training, the participants had to step as quickly as possible in different directions in response to the presentation of visual stimuli to gain points in the Fruits hunter and Where am I? games. Further, in the Whack a mole game, they had to step in different directions with different rule conditions. Such trainings might enhance the response ability and speed of processing. Improvement in postural sway was likely to be attributed to the postural stability required to play the Sky fall and Crossing poison river games. During these games, participants had to control their balance while standing on one leg, or while marching with a high knee.

In addition to the physiological fall risk parameter, which identified sensory motor impairment underlying falls, we also identified fall risk while performing functional mobility by using the TUG test. Although there was no significant difference in TUG time between the two groups, the intervention group had a significant reduction in the TUG time after training when compared to their baseline, suggesting a beneficial effect of training. Factors including task difficulty and participant characteristics might account for the non-significance in TUG time between the intervention and control groups. It might be possible that the TUG single test was not challenged enough to detect changes in relatively healthy, high-functioning older adults. This postulation was supported by their good TUG performance at baseline and was consistent with previous studies undertaken in similar samples [10,37]. Furthermore, the fact that there were significant group differences on TUG with the dual task suggests that this might be the case.

TUG with the dual task was used to indicate the dual-task ability. A faster TUG time while performing the verbal fluency reflected a better ability to divide attention, which was one dimension of the executive function. This finding is consistent with previous studies that reported the beneficial effect of simultaneously performing physical and cognitive training in improving the dual-task ability of older adults [10,11]. Dual-task ability might have been improved through an interactive physical-cognitive game-based training program in the way that participants had to concurrently divide their attention between physical and cognitive tasks. The dual-task paradigm has been widely used to demonstrate the interplay between mobility and cognition in both healthy older adults and those with cognitive impairment [5]. The finding of improved TUG dual-task performance suggests the beneficial effect of the combined physical-cognitive training on dual-task ability. The partial eta-squared (Table 1) suggested that the combined training had a medium effect on the TUG dual-task performance [38].

The present study also revealed the potential benefit of combined physical-cognitive training in improving global cognitive function as reflected by the MoCA score. The magnitude of improvement of MoCA score in the intervention group and the difference between the two groups exceeded the standard error of measurement (1.5 points) reported in a previous study [34]. Furthermore, the partial eta-squared (Table 1) indicated that the training had a large effect on the MoCA score [38]. Such improvement was mainly due to an increase in executive function, abstraction, and attention performance. Characteristics of the five games, which required selective attention to task-relevant, inhibition of task-irrelevant stimuli, divided attention, and planning/decision-making to achieve the game objective were likely to be responsible for the improvement in executive function and attention. Such a finding is consistent with those of previous studies [9,39,40] and supports the proposition that combined physical-cognitive training benefits cognitive performance. Although memory training was included in the program, the memory subtest of MoCA was not improved. This might be attributed to the short training duration of semantic memory. While executive function and attention were practiced through several games, semantic memory was practiced only in one game. Alternatively, it is also possible that MoCA might not be sensitive enough to detect memory changes, as it was designed to capture global cognitive performance [36]. On the one hand, the benefits of the interactive physical-cognitive, game-based training program on cognition might be due to the added cognitive training component, but on the other hand, it might be due to the synergistic effects of the combined training. Anderson-Hanley et al. [41] calculated the effect size of physical, cognitive, and combined physical-cognitive training on cognitive benefits. They found that the combined training had a medium effect size, whereas each single training (physical, cognitive) only had a small effect size, suggesting the synergistic effect of the combined physical-cognitive training. Further research is warranted to confirm this issue.

There are some limitations in this study. First, the study was not a randomized controlled trial and most participants recruited into the study were women (82.5%). Second, due to a lack of long-term follow-up, it is unknown whether the improvement shown in the experimental group can be sustained afterward. With these methodological limitations, further randomized controlled trial studies with equal gender distribution and long-term follow-up are warranted to confirm the study findings. Third, while the results demonstrated a statistically significant improvement for PPA, TUG dual task, and MoCA, interpretations on whether such improvements were real or due to measurement error and the clinically meaningful benefit of the training were not possible, due to unavailable data on the smallest real difference (SRD) and minimal clinically important difference (MCID) in these outcome measures. Finally, the participants in this study comprised reasonably healthy and high-functioning older adults, so findings might not be generalized to frail older people or those with health conditions.

## 5. Conclusions

The present study demonstrated that 12 weeks of the interactive physical-cognitive, game-based training program could reduce fall risk via improvement in the physiological fall risk factors including speed processing and body sway, improve global cognitive performance via improvement in executive function and attention, and improve dual-task performance. The interactive physical-cognitive, game-based training program could be practically implemented in community-dwelling older adults.

## Figures and Tables

**Table 1:** Summary of the cognitive domain included in the combined physical-cognitive training program.

Cognitive Domain	Training Purpose	Training Description
**Speed Processing** *Game 1:* Fruits hunter	To enhance response ability and speed of processing via stepping task	Step on the target presented as quickly as possible
**Memory & visuo-spatial** *Game 2:* Where am I?	To enhance semantic memory and visuo-spatial ability via visual sense	Stepping in concurrence with remembering the objects and its location presented Note: Delayed recall was assessed at the end of the game
**Executive function** **-inhibition** **-speed processing** **-attention** *Game 3:* Whack a mole	To improve selective attention, visual attention, speed of processing, and inhibition	Respond correctly to different rules of the game as quickly as possible Rules: - mole; step on the target 1 time - mole with helmet; step on the target twice - bat; step on the target 1 time - bat with helmet; step on the target twice - bomb; do not step on the target
**Sequencing & planning** *Game 4:* Sky fall	To improve sequencing and planning ability	Collect dropping objects into the basket. Several objects with different values (points) were dropping at the same time. The goal was to gain as high points as possible.
**Episodic Memory** *Game 5:* Crossing poison river	To improve episodic memory via auditory sense	Listen to a short story and remember the content of the story as much as possible while standing on one leg Note: Memory test (recall questions regarding the story’s content) was examined at the end of the game

**Table 2:** Participant demographic characteristics.

Characteristics	Intervention Group (*n* = 20)	Control Group (*n* = 20)	*p*-Value ^#^
Age (years)	70.21 ± 4.18	69.40 ± 3.38	0.509
Gender (male:female)	3:17	4:16	0.740
Height (cm)	155.95 ± 6.12	156.45 ± 7.34	0.819
Weight (kg)	57.45 ± 8.83	57.58 ± 9.48	0.964
Types of medication (n)	0.47 ± 0.70	0.70 ± 1.03	0.429
Falls in the past year (n)	5	4	0.699
Education (years)	12.79 ± 5.15	11.20 ± 4.80	0.325
MSET 10 (score)	26.26 ± 2.10	25.60 ± 2.90	0.424
TGDS (score)	1.16 ± 0.90	1.45 ± 1.57	0.479

Note: All values are expressed as means ± standard deviations except for gender. ^#^ Independent samples *t*-test (for continuous data) and chi-square test (for categorical data). Abbreviation: MSET10 = Mini-Mental State Examination T10 (total score = 29 points), TGDS = Thai Geriatric Depression Scale (total score = 15 points).

**Table 3:** Fall risk between the combined physical-cognitive training and control groups at baseline and the end of the 12 weeks.

Variables	Intervention Group (n = 19)		Control Group (n = 20)		Group × Time ^#^		
	Baseline	12-Week	Baseline	12-Week	F (1, 37)	*p*-Value	η_p_^2^
PPA Composite Score	0.79 ± 0.92	0.14 ± 0.79 ^a,b^	0.59 ± 0.70	0.81 ± 0.86	11.695	0.002	0.240
PPA sub-components							
Vision (dB)	20.74 ± 1.82	21.26 ± 1.28	19.95 ± 2.48	20.70 ± 2.90	0.128	0.722	0.003
Hand-reaction (ms)	264.02 ± 34 12	245.83 ± 31.13 ^b^	257.40 ± 37.30	268.74 ± 51.39	7.439	0.010	0.167
Postural sway (mm)	148.47 ± 63.76	106.53 ± 37 58 ^a,b^	118.70 ± 36.92	149.30 ± 50.29 ^b^	14.940	0.001	0.288
Proprioception (deg)	1.79 ± 0.86	1.85 ± 1.13	1.92 ± 0.89	1.47 ± 0.84	1.599	0.214	0.041
Knee extensor strength (kg)	22.70 ± 7.60	24.63 ± 4.17	22.75 ± 7.11	21.15 ± 5.96	3.749	0.061	0.092
TUG single task (s)	7.53 ± 0.10	6.86 ± 1.03 ^b^	7.61 ± 1.56	7.48 ± 1.31	6.510	**0.015**	0.150
TUG dual task (s)	9.57 ± 2.62	7.80 ± 1.50 ^a,b^	9.26 ± 2.60	8.96 ± 1.93	5.464	**0.025**	0.129

Note: All values are expressed as means ± standard deviations. ^#^ Analysis of two-way repeated-measures ANOVA; a = Significant difference between two groups, *p* < 0.05; b = Significant difference between baseline and post-training, *p* < 0.05.

**Table 4:** Cognitive performance between the combined physical-cognitive training and control groups at baseline and the end of the 12 weeks.

Variables	Intervention Group (n = 19)		Control Group (n = 20)		Group × Time ^#^		
	Baseline	12-week	Baseline	12-week	F (1, 37)	*p*-Value	η_p_^2^
MoCA (max. 30 points)	24.58 ± 3.53	26.37 ± 2.59 ^a,b^	24.40 ± 3.20	23.05 ±2.78 ^b^	15.743	0.001	0.298
MoCA subtests							
Executive (max. 5 points)	4.26 ± 0.65	4.68 ± 0.48 ^a^	4.05 ± 1.10	3.80 ± 1.10	4.722	0.036	0.113
Attention (max. 6 points)	5.05 ± 0.97	5.68 ± 0.58 ^a,b^	5.00 ± 1.12	4.50 ± 1.24 ^b^	14.692	0.001	0.284
Naming (max. 3 points)	2.95 ± 0.23	3.00 ± 0.00	2.95 ± 0.22	2.90 ± 0.31	2.001	0.166	0.051
Language (max. 3 points)	1.37 ± 1.15	1.74 ± 1.15 ^b^	1.40 ± 1.04	1.25 ± 0.91	4.620	0.038	0.111
Abstract (max. 2 points)	1.58 ± 0.61	1.68 ± 0.67 ^a^	1.45 ± 0.69	1.00 ± 0.86 ^b^	5.931	0.020	0.138
Memory (max. 5 points)	3.21 ± 1.81	3.47 ± 1.35	3.20 ± 1.82	3.20 ± 1.70	0.212	0.648	0.006
Orientation (max. 6 points)	5.89 ± 0.32	6.00 ± 0.00	5.95 ± 0.22	5.95 ± 0.22	1.054	0.311	0.028

Note: All values are expressed as means ± standard deviations. ^#^ Analysis of two-way repeated-measures ANOVA, a = Significant difference between two groups, *p* < 0.05; b = Significant difference between baseline and post-training, *p* < 0.05.
